# High glucose induces tau hyperphosphorylation in hippocampal neurons via inhibition of ALKBH5-mediated Dgkh m^6^A demethylation: a potential mechanism for diabetic cognitive dysfunction

**DOI:** 10.1038/s41419-023-05909-7

**Published:** 2023-06-29

**Authors:** Minli Qu, Linhui Zuo, Mengru Zhang, Peng Cheng, Zhanjun Guo, Junya Yang, Changjun Li, Jing Wu

**Affiliations:** 1 Department of Endocrinology, Xiangya Hospital, Central South University, Changsha, Hunan China; 2 School of Health and Related Research, University of Sheffield, Sheffield, UK; 3 Endocrinology Research Center, Xiangya Hospital, Central South University, Changsha, Hunan China; 4 Hunan Engineering Research Center for Obesity and its Metabolic Complications, Xiangya Hospital, Central South University, Changsha, Hunan China; 5 National Clinical Research Center for Geriatric Disorders, Xiangya Hospital, Central South University, Changsha, China

**Keywords:** Cognitive control, Diabetes complications

## Abstract

Tau hyperphosphorylation in hippocampal neurons has an important pathogenetic role in the development of diabetic cognitive dysfunction. N^6^-methyladenosine (m^6^A) methylation is the most common modification of eukaryotic mRNA and is involved in regulating diverse biological processes. However, the role of m^6^A alteration in tau hyperphosphorylation of hippocampus neurons has not been reported. We found lower ALKBH5 expression in the hippocampus of diabetic rats and in HN-h cells with high-glucose intervention, accompanied by tau hyperphosphorylation. ALKBH5 overexpression significantly reversed tau hyperphosphorylation in high-glucose-stimulated HN-h cells. Furthermore, we found and confirmed by m^6^A–mRNA epitope transcriptome microarray and transcriptome RNA sequencing coupled with methylated RNA immunoprecipitation that ALKBH5 regulates the m^6^A modification of Dgkh mRNA. High glucose inhibited the demethylation modification of Dgkh by ALKBH5, resulting in decreases in Dgkh mRNA and protein levels. Overexpression of Dgkh reversed tau hyperphosphorylation in HN-h cells after high-glucose stimulation. Overexpression of Dgkh by adenovirus suspension injection into the bilateral hippocampus of diabetic rats significantly ameliorated tau hyperphosphorylation and diabetic cognitive dysfunction. In addition, ALKBH5 targeted Dgkh to activate PKC-α, leading to tau hyperphosphorylation under high-glucose conditions. The results of this study reveal that high glucose suppresses the demethylation modification of Dgkh by ALKBH5, which downregulates Dgkh and leads to tau hyperphosphorylation through activation of PKC-α in hippocampal neurons. These findings may indicate a new mechanism and a novel therapeutic target for diabetic cognitive dysfunction.

## Introduction

Diabetes mellitus (DM) is a common chronic disease characterized by elevated blood glucose and chronic complications affecting all vital organs throughout the body. Diabetes can affect the central nervous system, and the prevalence of cognitive dysfunction or Alzheimer’s disease (AD) in DM patients is two to three times higher than that in control subjects [1–3]. The decline in cognitive function has a significant impact on a patient’s quality of life and on the implementation of treatment plans [4].

The hippocampus is the center of cognition and memory and is an important brain region for maintaining cognitive function [5, 6]. Tau protein is the most abundant microtubule-associated protein and has a role in maintaining microtubule stability in neuronal cells [7]. In pathological states, tau hyperphosphorylation is among the important changes causing neurodegenerative diseases [8]. High glucose has been identified as one of the major causes of tau hyperphosphorylation in hippocampal neurons, which mediates cognitive dysfunction in diabetes [9–11]. Hence, it is critical to investigate the mechanism by which tau protein hyperphosphorylation is induced by high glucose (HG).

N^6^-methyladenosine (m^6^A), an abundant post-transcriptional modification of most eukaryote mRNAs, is the most prevalent mRNA modification in mammalian cells [12]. m^6^A modification has been implicated in RNA fate including mRNA splicing, nuclear export, and translation [13–15]. The “writer” m^6^A methyltransferases, which include methyltransferase-like 3 (METTL3) and METTL14, perform the modification reaction, whereas the “eraser” m^6^A demethylases, which include fat mass and obesity-associated protein (FTO) and AlkB homolog 5 (ALKBH5), reverse this modification [16]. m^6^A modification is highly abundant in the nervous system [17], and m^6^A-related enzymes influence hippocampus-dependent learning and memory [18]. Studies have revealed associations of m^6^A with central nervous system diseases including AD [19, 20], traumatic brain injury [21, 22], and Parkinson’s disease (PD) [23, 24]. ALKBH5 is a well-known m^6^A demethylase that is mainly localized in nuclear speckles [25]. ALKBH5 has a crucial role in both neurodevelopment and neurodegenerative diseases [23, 26]. In ALKBH5-deficient mice, cell proliferation and differentiation in the cerebellum are impaired, and whole brain volume is reduced [26]. In addition, recent studies have reported that m^6^A modifications are strongly associated with diabetes and its related complications [27–30]. There have also been preliminary reports of changes in m^6^A-related enzymes in diabetic cognitive dysfunction [30]. However, the role of m^6^A demethylase ALKBH5 in diabetic cognitive dysfunction and the mechanism of tau hyperphosphorylation remain unknown.

In this study, we demonstrate the function of ALKBH5 in facilitating tau phosphorylation in hippocampal neurons and identify diacylglycerol kinase eta (Dgkh) as the downstream target of ALKBH5 in diabetic cognitive dysfunction by m^6^A-mRNA epitope transcriptome microarray and transcriptome RNA sequencing (RNA-seq). We thus reveal a potential new predictive biomarker and therapeutic target for reversing diabetic cognitive dysfunction.

## Results

### Tau hyperphosphorylation in diabetic rats is accompanied by decreased expression of m^6^A demethylase ALKBH5

**Fig. 1:**
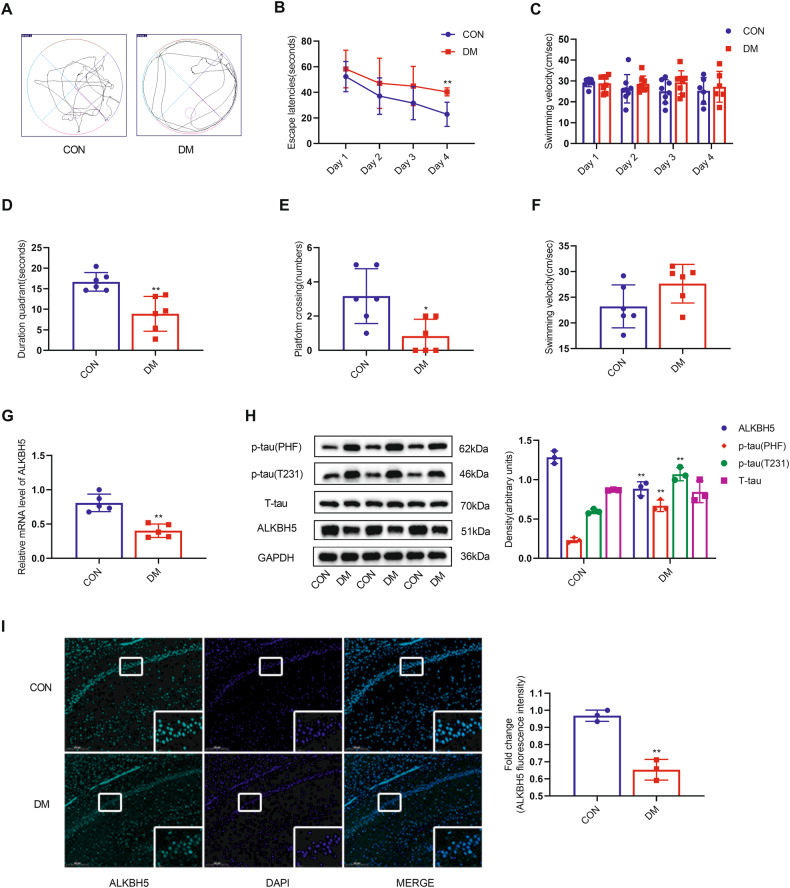
Diabetic rats show cognitive dysfunction with downregulation of ALKBH5 expression in hippocampal neurons. **A**–**F** MWM test of control and diabetic rats (*n* = 6 for CON group, *n* = 8 for DM group). **A** Representative swimming traces during the space exploration phase. **B** Escape latency during training. **C** Swimming velocity during training. **D** Escape latency during the space exploration phase. **E** Numbers of platform crossings during the space exploration phase. **F** Swimming velocity during the space exploration phase. **G** Real-time quantitative PCR analysis of ALKBH5 mRNA expression (*n* = 4). **H** Western blots and quantitative densitometry analysis of ALKBH5, p-tau (T231), p-tau (PHF), and T-tau (*n* = 3). **I** Representative images and quantitative analysis of ALKBH5 intensities for paraffin-embedded sections from the CON group and the DM group (*n* = 3). Scale bar, 200 μm. Student’s two-sample *t*-test and Mann–Whitney test were used to detect differences between the two groups. *p < 0.05, **p < 0.01 vs CON. Error bars represent s.e.m.

In diabetic rats, fasting blood glucose levels increased sharply from day 3 after streptozotocin (STZ) injection and remained consistently elevated at week 12 week compared with the control (CON) group (Table 1). After 12 weeks post STZ injection, in the probing test, the diabetic rats showed a limbic movement pattern, in contrast to the CON group, indicating that the diabetic rats only retained inaccurate spatial memory (Fig. 1A). The average escape latency of rats in both the DM and CON groups decreased as the number of training days increased, and diabetic rats took longer to get on the platform on training day 4 compared with CON rats (Fig. 1B). In addition, compared with the CON group, the diabetic rats spent significantly less time in the target quadrant of the hidden platform and traversed the platform more often (Fig. 1D, E). No significant difference in swimming speed was observed between the diabetic and CON groups (Fig. 1C, F). Our findings suggest that STZ-induced diabetic rats have impaired spatial learning memory capacity.

**Table 1:** The changes in blood glucose levels in the experimental groups.

Groups	Control group	DM group
Number of rats	8	8
FBG 3 days after STZ injected (mM)	5.77 ± 0.87	27.98 ± 4.09**
FBG 12 weeks (mM)	5.83 ± 0.49	22.29 ± 3.4**

*DM* Diabetes mellitus, *FBG* fasting blood glucose, *STZ* streptozotocin. Compared with the control group, fasting blood glucose was significantly higher in the DM group, and the difference was statistically significant (**p < 0.01).

To clarify the role of m^6^A modification in diabetic cognitive dysfunction, we measured the mRNA (Fig. S1A) and protein (Fig. S1B) levels of four core enzymes of m^6^A modification, METTL3, METTL14, FTO, and ALKBH5, in hippocampal tissues. We found that only m^6^A demethylase ALKBH5 had significantly decreased mRNA and protein levels in the diabetic rat group compared with the CON group. The other m^6^A modification-related enzymes showed no significant changes.

Tau hyperphosphorylation is an important pathological change leading to cognitive dysfunction in diabetes. We found that decreased ALKBH5 expression (Fig. 1G, H) was accompanied by increased tau hyperphosphorylation. In the hippocampus of diabetic rats, p-tau levels (but not total tau levels) were significantly increased in both Thr231 (T231) and Ser396/404 (PHF-1) epitopes. Furthermore, decreased ALKBH5 positive signals were observed in hippocampal CA1 pyramidal neurons of the diabetic brain compared with the CON group, as demonstrated by immunofluorescence staining (Fig. 1I). Taken together, the above results show that the impairment of spatial learning and memory in STZ-induced diabetes rats is accompanied by downregulation of m^6^A demethylase ALKBH5.

### High glucose promotes tau hyperphosphorylation by inhibiting the expression of m^6^A demethylase ALKBH5 in HN-h cells

**Fig. 2:**
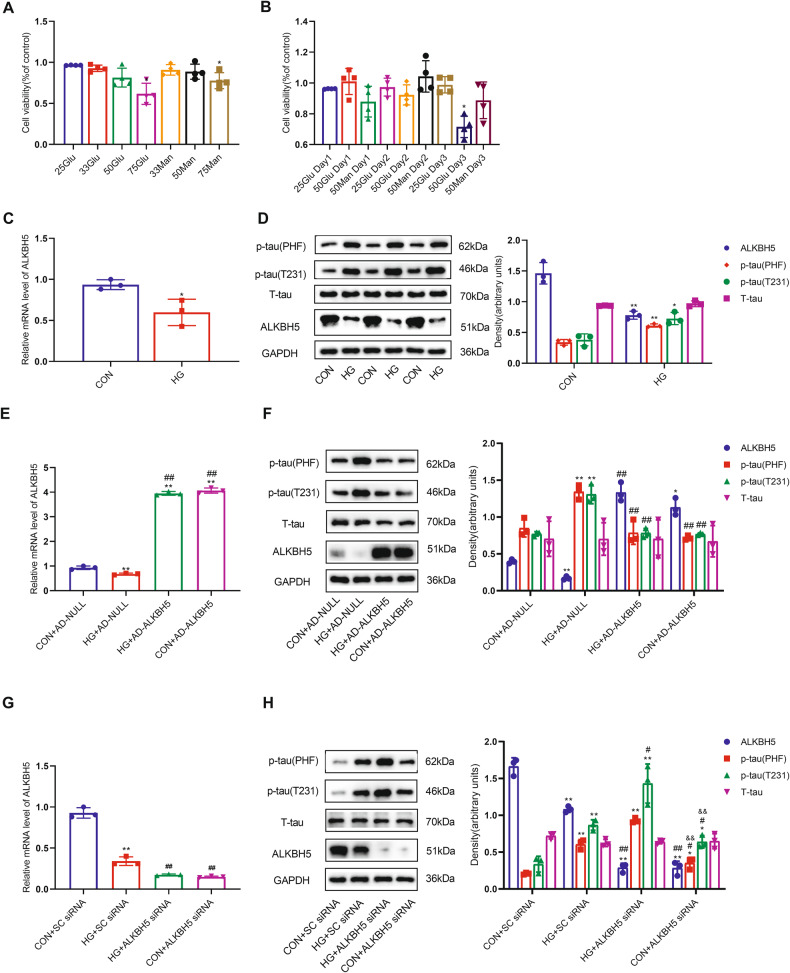
ALKBH5 downregulation leads to tau hyperphosphorylation in HN-h cells. **A** Effects of different concentrations of glucose and mannitol on the viability of HN-h cells (*n* = 3). **B** Effects of different intervention times with 50 mM glucose and 50 mM mannitol on the viability of HN-h cells (*n* = 3). **C** Real-time quantitative PCR analysis of ALKBH5 mRNA expression; and **D** Western blots and quantitative densitometry analysis of ALKBH5, p-tau (T231), p-tau (PHF), and T-tau in HN-h cells with 50 mM glucose (*n* = 3). Student’s two-sample *t*-test and Mann–Whitney test were used to detect differences between the two groups. *p < 0.05, **p < 0.01 vs CON. **E** Real-time quantitative PCR of ALKBH5; and **F** western blots and quantitative densitometry analysis of ALKBH5, p-tau (T231), p-tau (PHF), and T-tau in HN-h cells overexpressing ALKBH5 using lentivirus (*n* = 3). *p < 0.05, **p < 0.01 vs Con+AD-NULL. ^#^*p* < 0.05, ^##^*p* < 0.01 vs HG + AD-NULL. **G** Real-time quantitative PCR analysis of ALKBH5; and **H** western blots and quantitative densitometry analysis of ALKBH5, p-tau (T231), p-tau (PHF), and T-tau in HN-h cells with knockdown of ALKBH5. One-way ANOVA and Tukey–Kramer test were used to detect differences among groups. *p < 0.05, **p < 0.01 vs CON + SC siRNA. ^#^*p* < 0.05, ^##^*p* < 0.01 vs HG + SC siRNA. ^&^*p* < 0.05, ^&&^*p* < 0.01 vs HG + ALKBH5 siRNA. Error bars represent s.e.m.

We exposed Human Neurons-hippocampal (HN-h) cells to different HG concentrations (25, 33, 50, and 75 mM) with different intervention times to determine suitable conditions that did not affect cell viability. We found a significant decrease in cell viability with 75 mM glucose intervention for 2 days compared with the CON group (Fig. 2A). Cell viability was also reduced after cells had been treated with 50 mM glucose for 3 days (Fig. 2B), whereas treatment with an osmolarity control (25 mM glucose plus 50 mM mannitol) for 3 days did not significantly affect cell viability. Consequently, we chose 50 mM glucose treatment for 2 days for the subsequent experiments. We found that both the mRNA and protein levels of ALKBH5 were significantly reduced in the HG group compared with normal controls (Fig. 2C, D). There were no significant changes in the mRNA and protein levels (Fig. S2A, B) of METTL3, METTL14, or FTO. In addition, the p-tau levels of the T231 and PHF-1 epitopes were significantly increased after HG treatment (Fig. 2D). These results suggest that HG results in tau hyperphosphorylation in HN-h cells accompanied by downregulation of ALKBH5 expression.

To further confirm the involvement of ALKBH5 in regulating tau phosphorylation, we infected HN-h cells with a lentivirus encoding ALKBH5 (Fig. 2E). This overexpression of ALKBH5 reversed the tau hyperphosphorylation caused by HG (Fig. 2F). In addition, silencing ALKBH5 with a small interfering RNA (siRNA) (Fig. 2G) in HN-h cells resulted in a slight increase in p-tau levels of the T231 and PHF-1 epitopes compared with the control. Compared with the HG + SC (scrambled) siRNA group, the p-tau levels of the T231 and PHF-1 epitopes were more significantly increased in the HG + ALKBH5 siRNA group (Fig. 2H). These results suggest that HG decreases the expression of m^6^A demethylase ALKBH5 and thus leads to tau protein hyperphosphorylation.

### Overview of differentially methylated mRNAs analysis and RNA-seq in hippocampal of diabetic rats

**Fig. 3:**
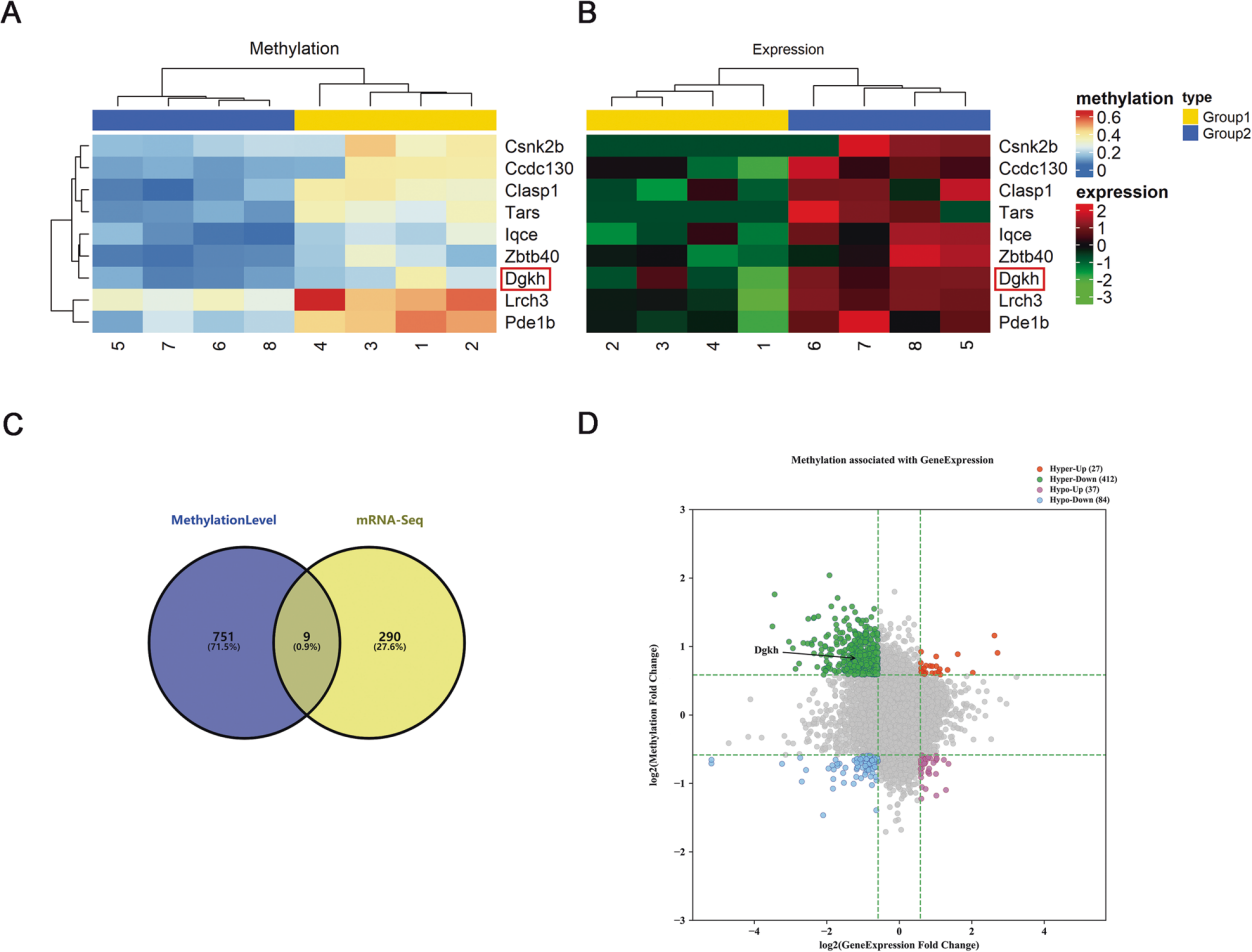
Overview of altered m^6^A methylation maps in the hippocampus of diabetic rats. **A** Clustering map of the m^6^A-mRNA Arraystar, V1.0 and **B** clustering map of mRNA sequencing. The analysis revealed that nine genes were changed with respect to both epitranscriptional modification level and mRNA transcription level of m^6^A (*p* ≤ 0.05, fold change > 1.5), (1–4 are DM group and 5–8 are CON group). **C** Venn diagram. **D** Quadrant chart (*n* = 4).

Under HG conditions, downregulation of m^6^A demethylase ALKBH5 expression in the hippocampus causes an increase in levels of m^6^A modification. Therefore, we performed an m^6^A-mRNA epitope transcriptome microarray (Arraystar, V1.0) assay on hippocampal tissues of DM and normal rats to identify genes with upregulation of m^6^A modifications. We also performed RNA-seq on hippocampal tissues of DM and normal rats to identify differentially expressed genes. Then, the results of the two experiments were analyzed jointly, and nine genes were found to be differentially altered with respect to both epistatic transcriptional modification levels and mRNA transcripts level of m^6^A (*p* ≤ 0.05, fold change > 1.5). The nine genes were charted using a heat map (Fig. 3A, B), and a Venn diagram was generated (Fig. 3C). In the figure, the four-quadrant plot in the upper left (green) represents 412 differential genes, including Dgkh, with increased m^6^A modification levels and decreased mRNA levels in the DM group compared with the CON group. In the lower left, blue represents 84 differential genes with decreased m^6^A modification levels and decreased mRNA levels; in the upper right, red represents 27 differential genes with increased m^6^A modification levels; and in the lower right, purple represents 37 differential genes with decreased m^6^A modification levels and increased mRNA levels (Fig. 3D).

### High glucose inhibits m^6^A demethylation modification of Dgkh by ALKBH5, leading to decreased Dgkh levels

**Fig. 4:**
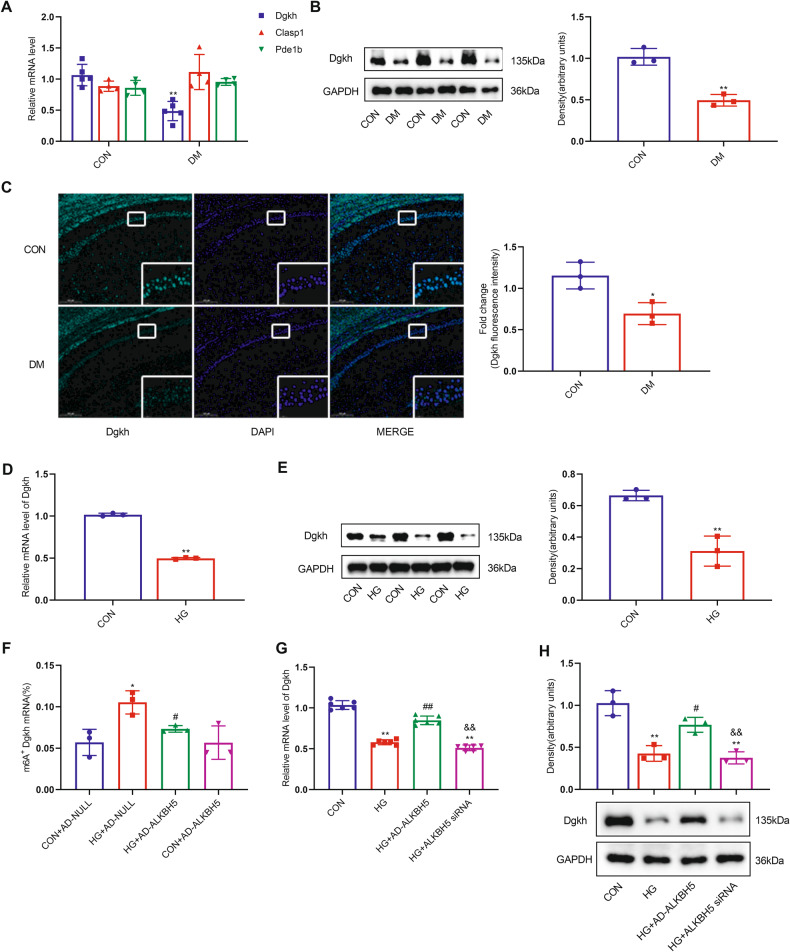
ALKBH5 demethylates Dgkh, leading to downregulation of Dgkh expression under hyperglycemic conditions. **A** Real-time quantitative PCR analysis of Dgkh, Clasp1, and Pde1b mRNA expression in the hippocampus of diabetic rats (*n* = 3). **B** Western blots and quantitative densitometry analysis of Dgkh protein expression in the hippocampus of diabetic rats (*n* = 3). **C** Representative images and quantitative analysis of Dgkh intensities for paraffin-embedded sections from the CON group and the DM group. Scale bar, 200 μm (*n* = 3). Student’s two-sample *t*-test and Mann–Whitney test were used to detect differences between the two groups. *p < 0.05, **p < 0.01 vs CON. **D** Real-time quantitative PCR analysis of Dgkh mRNA expression; and **E** western blots and quantitative densitometry analysis of Dgkh in HN-h cells with 50 mM glucose (*n* = 3). Student’s two-sample *t*-test and Mann–Whitney test were used to detect differences between the two groups. *p < 0.05, **p < 0.01 vs CON. **F** m^6^A immunoprecipitation and real-time quantitative PCR were performed to determine the percentage of Dgkh mRNA with methylation (*n* = 3). One-way ANOVA and Tukey–Kramer test were used to detect differences among groups. *p < 0.05 vs CON + AD-NULL, ^#^*p* < 0.05 vs HG + AD-NULL. **G** Real-time quantitative PCR analysis of Dgkh mRNA expression; and **H** western blots and quantitative densitometry analysis of Dgkh in HN-h cells with ALKBH5 knockdown or ALKBH5 overexpression (*n* = 3). One-way ANOVA and Tukey–Kramer test were used to detect differences among groups. **p < 0.01 vs CON, ^#^*p* < 0.05, ^##^*p* < 0.01 vs HG, ^&&^*p* < 0.01 vs HG + AD-ALKBH5. Error bars represent s.e.m.

By analyzing our data and reviewing the literature, we found that Dgkh, Clasp1 (cytoplasmic linker-associated protein 1), and Pde1b (phosphodiesterase 1b) are closely related to central nervous system disease. According to the subsequent quantitative PCR validation, only Dgkh could be verified to be differentially expressed in hippocampal tissues between the diabetic and normal groups (Fig. 4A). We also found that the protein level of Dgkh was significantly downregulated in the DM group compared with the normal CON group (Fig. 4B). In addition, immunofluorescence staining results showed that the immunoreactivity of Dgkh was significantly diminished in hippocampal CA1 pyramidal neurons of the diabetic brain compared with normal controls (Fig. 4C). Similarly, the expression of both mRNA and protein levels of Dgkh was significantly downregulated in the HG state in the cell model (Fig. 4D, E).

To confirm that the m^6^A modification of Dgkh mRNA is regulated by ALKBH5, we demonstrated by MeRIP-qRT-PCR that HG did indeed lead to an increase in m^6^A methylation modification of Dgkh mRNA, which could be significantly reversed by overexpression of ALKBH5 in HN-h cells with HG stimulation (Fig. 4F). Furthermore, overexpression of ALKBH5 effectively reversed the decrease in Dgkh expression caused by HG (Fig. 4G, H). These results suggest that demethylated ALKBH5 affects the protein expression level of Dgkh by regulating the m^6^A modification of Dgkh under HG conditions.

### High glucose downregulates Dgkh expression, leading to tau protein hyperphosphorylation

**Fig. 5:**
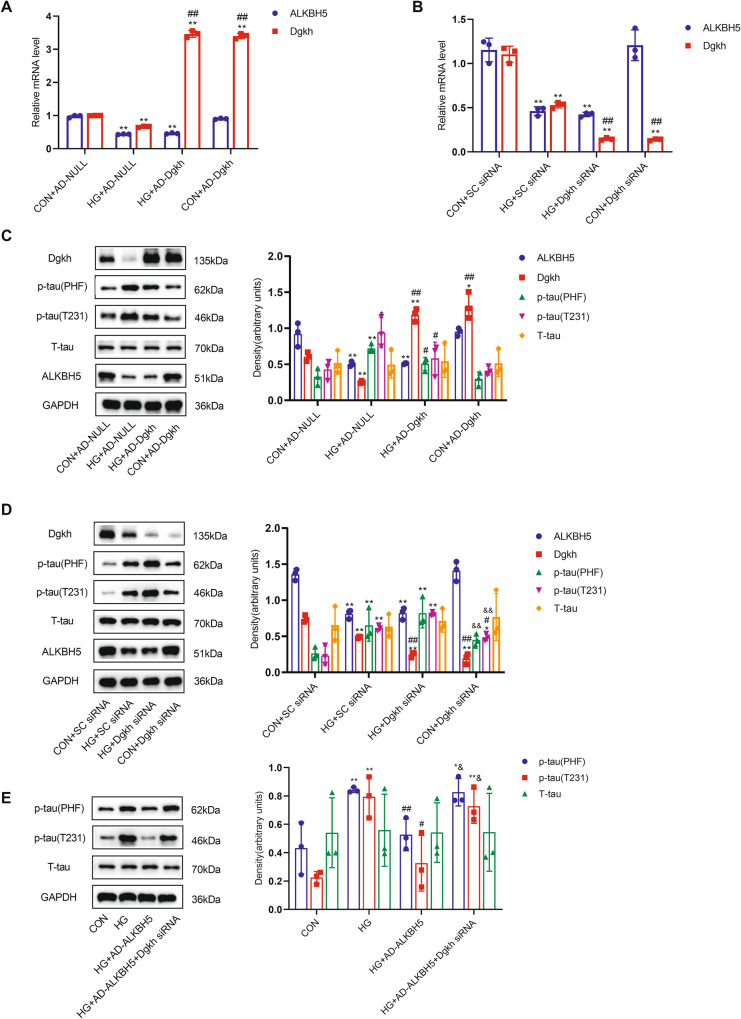
Decreased Dgkh contributes to the formation of tau hyperphosphorylation under hyperglycemic conditions. **A** Real-time quantitative PCR analysis of ALKBH5 and Dgkh in HN-h cells overexpressing Dgkh using adenovirus (*n* = 3). **B** Real-time quantitative PCR analysis of ALKBH5 and Dgkh in HN-h cells with knockdown of Dgkh (*n* = 3). **C** Western blots and quantitative densitometry analysis of Dgkh, p-tau (T231), p-tau (PHF), and T-tau in HN-h cells overexpressing Dgkh using adenovirus (*n* = 3). **D** Western blots and quantitative densitometry analysis of Dgkh, p-tau (T231), p-tau (PHF), and T-tau in HN-h cells with knockdown of Dgkh (*n* = 3). One-way ANOVA and Tukey–Kramer test were used to detect differences among groups. *p < 0.05, **p < 0.01 vs CON + AD-NULL or CON + SC siRNA; ^#^*p* < 0.05, ^##^*p* < 0.01 vs HG^+^AD-NULL or HG + SC siRNA; ^&^*p* < 0.05, ^&&^*p* < 0.01 vs HG+Dgkh siRNA. **E** Western blots and quantitative densitometry analysis of p-tau (T231), p-tau (PHF), and T-tau in HN-h cells with ALKBH5 overexpression and Dgkh knockdown. One-way ANOVA and Tukey–Kramer test were used to detect differences among groups. *p < 0.05, **p < 0.01 vs CON; ^#^*p* < 0.05, ^##^*p* < 0.01 vs HG; ^&^*p* < 0.05, ^&&^*p* < 0.01 vs HG + AD-ALKBH5. Error bars represent s.e.m.

Overexpression of Dgkh in HN-h cells did not affect the mRNA level of ALKBH5 (Fig. 5A). As shown in Fig. 5C, overexpression of Dgkh in HN-h cells reversed the HG-induced significant reduction in p-tau levels of T231 and PHF-1 epitopes without affecting the expression of ALKBH5. In addition, silencing of Dgkh with siRNA in HN-h cells (Fig. 5B) resulted in a slight increase in p-tau of T231 and PHF-1 epitopes compared with the normal control group transfected with SC-siRNA. The increases in p-tau levels of T231 and PHF-1 epitopes were more pronounced in the HG+Dgkh siRNA group compared with the HG + SC siRNA group (Fig. 5D). Overexpression of ALKBH5 in HG-treated HN-h cells reversed tau protein hyperphosphorylation, in contrast to the HG + AD-NULL group, whereas p-tau levels of both T231 and PHF-1 epitopes remained increased after overexpression of ALKBH5 with concomitant silencing of Dgkh compared with those of the HG + AD-ALKBH5 group (Fig. 5E). These results suggest that HG downregulates Dgkh expression leading to tau hyperphosphorylation and that overexpression of ALKBH5 reverses HG-induced tau hyperphosphorylation in HN-h cells in a manner requiring the involvement of Dgkh.

### Overexpression of Dgkh improves cognitive dysfunction in diabetic rats

**Fig. 6:**
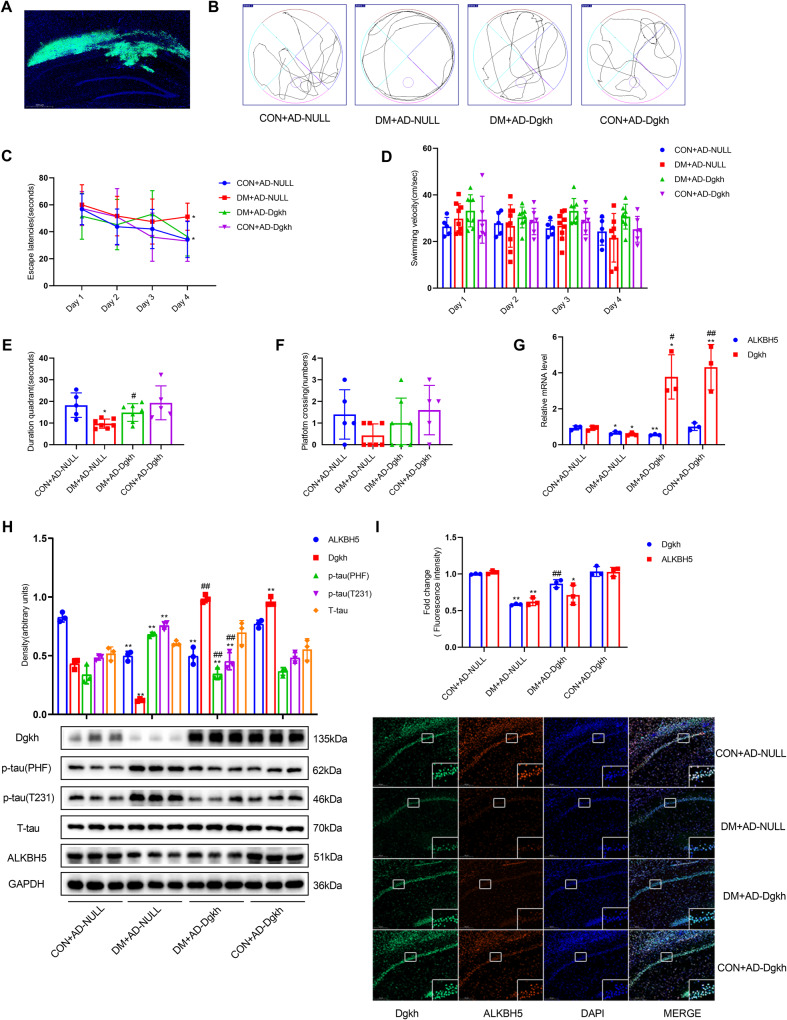
Overexpression of Dgkh alleviates cognitive dysfunction in diabetic rats. Adenovirus-mediated overexpression of Dgkh was injected into the hippocampal CA1 region by stereotaxic injection (*n* = 5 for CON + AD-NULL group, *n* = 8 for DM + AD-NULL group, *n* = 8 for AD + AD-Dgkh group, *n* = 6 for CON + AD-Dgkh group). **A** Diagram of viral infusion of Dgkh-overexpression constructs into the CA1. Scale bar, 200 μm. **B** Representative swimming traces during the space exploration phase. **C** Escape latency during training. **D** Swimming velocity during training. **E** Escape latency during the space exploration phase. **F** Numbers of platform crossings during the space exploration phase. **G** Real-time quantitative PCR analysis of ALKBH5 and Dgkh mRNA expression (*n* = 3). **H** Western blots and quantitative densitometry analysis of ALKBH5, Dgkh, p-tau (T231), p-tau (PHF), and T-tau (*n* = 3). **I** Representative images and quantitative analysis of Dgkh and ALKBH5 intensities for paraffin-embedded sections from four groups of rats (*n* = 3). Dgkh is shown in green. ALKBH5 is shown in red. Scale bar, 200 μm. One-way ANOVA, Tukey–Kramer test, and Kruskal–Wallis H test was used to detect differences among groups. *p < 0.05, **p < 0.01 vs Con+AD-NULL; ^#^*p* < 0.05, ^##^*p* < 0.01 vs HG^+^AD-NULL. Error bars represent s.e.m.

Next, we investigated whether overexpression of Dgkh within the CA1 region could rescue cognitive dysfunction in diabetic animals. We injected adenovirus overexpressing Dgkh into the CA1 region of the hippocampus bilaterally in diabetic rats. Dgkh was successfully overexpressed in the CA1 region of the hippocampus, as demonstrated by immunofluorescence staining (Fig. 6A). We observed no differences in the body weights and blood glucose levels of Dgkh-overexpression rats compared with CON + AD-NULL rats, as shown in Table 2. Of note, overexpressing Dgkh in the CA1 region dramatically reduced the proportion of marginal circling movements compared with those of the rats that received a vector adenovirus injection (Fig. 6B). Rats in the DM + AD-Dgkh group took shorter to get on the platform on training day 4 than the DM + AD-NULL group rats (Fig. 6C). Dgkh-overexpression rats also spent significantly more time in the target quadrant of the hidden platform (Fig. 6E). Compared with the diabetes group, there was a tendency for diabetic rats overexpressing Dgkh to cross the plateau more often (Fig. 6F). No difference was observed in the swimming speed between the DM and control groups (Fig. 6D).

**Table 2:** The changes in blood glucose levels in the experimental groups.

Groups	CON + AD-NULL	DM + AD-NULL	DM + AD-Dgkh	CON + AD-Dgkh
Number of rats	8	8	8	8
FBG 3 days after STZ injected (mM)	5.74 ± 0.79	28.06 ± 3.36**	26.8 ± 4.76**	5.85 ± 0.82
FBG 12 weeks (mM)	6 ± 0.23	23.94 ± 3.61**	23.96 ± 2.96**	5.7 ± 0.43

*DM* Diabetes mellitus, *FBG* fasting blood glucose, *STZ* streptozotocin. Compared with the CON + AD-NULL group, fasting blood glucose was significantly higher in the DM + AD-NULL and the DM + AD-Dgkh group, and the difference was statistically significant (**p < 0.01). There is no difference in fasting blood glucose between the DM + AD-NULL and the DM + AD-Dgkh group.

Further, we examined the p-tau levels at the T231 and PHF-1 epitopes and found that tau phosphorylation levels were significantly lower in hippocampal tissues after overexpression of Dgkh compared with the DM + AD-NULL group (Fig. 6H). By detecting the mRNA and protein levels of ALKBH5 and Dgkh in hippocampal tissues, we confirmed the successful overexpression of Dgkh. ALKBH5 expression remained decreased in diabetic rats, and overexpression of Dgkh had no effect on the expression of ALKBH5 (Fig. 6G–I). These results suggest that overexpression of Dgkh in the hippocampus can improve cognitive dysfunction in diabetic rats.

### ALKBH5 targets Dgkh to activate PKC-α, leading to tau hyperphosphorylation under HG conditions

**Fig. 7:**
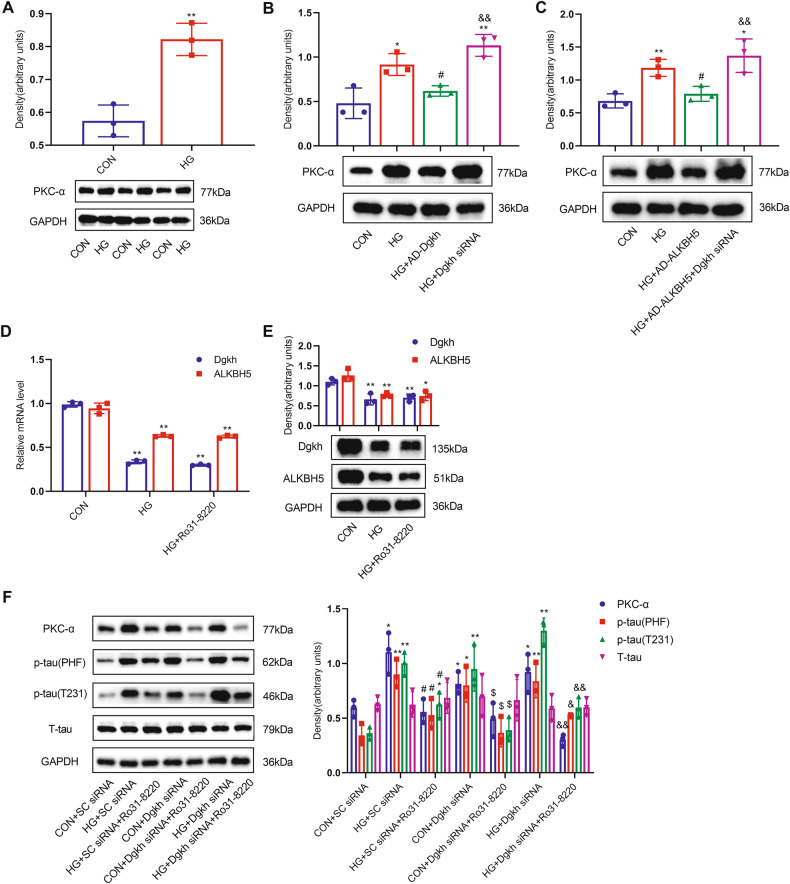
ALKBH5 targets Dgkh to activate PKC-α, leading to tau hyperphosphorylation under HG conditions. **A** Western blots and quantitative densitometry analysis of PKC-α in HN-h cells with 50 mM glucose (*n* = 3). Student’s two-sample *t*-test and Mann–Whitney test were used to detect differences between the two groups. **p < 0.01 vs CON. **B** Western blots and quantitative densitometry analysis of PKC-α in HN-h cells with Dgkh overexpression and/or knockdown (*n* = 3). One-way ANOVA and Tukey–Kramer test were used to detect differences among groups. *p < 0.05, **p < 0.01 vs CON; ^##^*p* < 0.01 vs HG; ^&&^*p* < 0.01 vs HG + AD-Dgkh. **C** Western blots and quantitative densitometry analysis of PKC-α in HN-h cells with ALKBH5 overexpression and/or Dgkh knockdown (*n* = 3). *p < 0.05, **p < 0.01 vs CON; ^#^*p* < 0.05 vs HG; ^&&^*p* < 0.01 vs HG + AD-ALKBH5. **D**, **E** mRNA and protein expression of ALKBH5 and Dgkh in HN-h cells pretreated with PKC-α inhibitor Ro31-8220 (*n* = 3). Student’s two-sample *t*-test and Mann–Whitney test were used to detect differences between the two groups. *p < 0.05, **p < 0.01 vs CON. **F** PKC-α is required for HG and/or Dgkh knockdown-induced tau hyperphosphorylation (*n* = 3). One-way ANOVA and Tukey–Kramer test were used to detect differences among groups. *p < 0.05, **p < 0.01 vs CON + SC siRNA; ^#^*p* < 0.05, vs HG + SC siRNA; ^$^*p* < 0.05 vs CON+ Dgkh siRNA; ^&^*p* < 0.05, ^&&^*p* < 0.01 vs HG+ Dgkh siRNA. Error bars represent s.e.m.

To further characterize the role of Dgkh in diabetic cognitive dysfunction, we determined the expression of protein kinase C-α (PKC-α). PKC-α is regulated by DGKs and can phosphorylate tau, as previously reported [31–33]. We observed significantly increased PKC-α expression in the HG group compared with normal controls (Fig. 7A). HN-h cells transfected with Dgkh siRNA showed significantly higher levels of PKC-α under HG stimulation. By contrast, HN-h cells with Dgkh overexpression had lower levels of PKC-α compared with those in the HG group (Fig. 7B). ALKBH5 overexpression ameliorated the increase in PKC-α expression under HG conditions. Compared with ALKBH5-overexpressing cells, HN-h cells with Dgkh siRNA had higher levels of PKC-α under HG conditions (Fig. 7C). Levels of ALKBH5 and Dgkh showed no changes after treatment with PKC-α inhibitor Ro31-8220 compared with the HG group, suggesting that the expression of ALKBH5 and Dgkh is not regulated by PKC-α (Fig. 7D, E). When HN-h cells were pretreated with PKC-α inhibitor Ro31-8220, the tau hyperphosphorylation induced by HG and/or Dgkh knockdown disappeared (Fig. 7F). In addition, we found that the level of PKC-α in diabetic hippocampal tissues was significantly increased compared with that of the CON group (Fig. S3A). PKC-α levels were significantly reduced in hippocampal tissues after overexpression of Dgkh compared with the DM + AD-NULL group (Fig. S3B). Based on these results, we conclude that Dgkh may promote tau phosphorylation by activating PKC-α.

## Discussion

Several studies have reported that tau hyperphosphorylation-induced neurodegeneration is the key pathological feature of diabetic cognitive dysfunction [9, 34–36]. Tau is a primarily neuronal protein comprising two major domains that contain multiple Thr-Pro and Ser-Pro motifs: a projection domain (for example, S181, S202/T205 (AT8), T212/S214 (AT100), and T231) and a microtubule assembly domain (for example, S262, S356 and S396/S404 (PHF-1)) [37]. Planel et al. observed highly phosphorylated proteins, including p-tau (AT8), p-tau (PHF-1), and p -tau (S262), in hippocampal tissue from day 40 after STZ injection; these were mainly distributed in axons and nerve fiber networks of neurons, consistent with the degree of cognitive dysfunction [38]. In another study, there was an increase in tau phosphorylation at the S396 and S181 epitopes in response to STZ-induced diabetic rats at 12 weeks [39]. In the present study, we observed significantly higher p-tau (T231) and p-tau (PHF-1) levels in the hippocampus of STZ-induced diabetic rats at 12 weeks. These findings were consistent with our previous research [11]. The degree of cognitive impairment is positively associated with tau hyperphosphorylation in hippocampal neurons.

m^6^A modification of mRNAs or non-coding RNAs is involved in various cellular processes, regulates RNA fate and functions, modulates essential life processes such as normal physiology and abnormal pathology, and has become a hot topic in the DM field in recent years [40]. FTO and ALKBH5 are known as m^6^A “erasers” [41, 42], whereas METTL3 and METTL14 are referred to as m^6^A “writers” [43, 44]. We found that demethylase ALKBH5 was significantly reduced in the hippocampal tissues of diabetic rats, whereas the expression of the other enzymes (METTL3, METTL14, and FTO) was not significantly altered. Song et al. reported cognitive dysfunction and hippocampal neuronal apoptosis after 14 days in STZ-induced diabetic rats, as well as significant increases in METTL3 and METTL14 and decreases in FTO and ALKBH5 expression in hippocampal tissues, but they did not identify any change in tau phosphorylation or the function of methylation enzymes. We speculate that the discrepancy with our data is due to the difference in the timing of STZ intervention. m^6^A-modifying enzymes may change with the duration of DM. Several studies have reported that levels of m^6^A methylesterase in DM vary over time [45]. ALKBH5 is mainly localized in nuclear speckles and has been shown to have a crucial role in both neurodevelopmental and neurodegenerative diseases. ALKBH5 deletion impaired neural progenitor cell proliferation and differentiation and reduced whole-brain volume [26]. Increased ALKBH5 expression in the striatum of 6-OHDA-induced PD rats resulted in lower m^6^A levels, which may accelerate excitotoxic cell death of dopaminergic neurons [23]. Five m^6^A single-nucleotide polymorphisms associated with PD have been identified in PD patients, three of which are in the ALKBH5 gene [24]. In our study, we found reduced ALKBH5 expression accompanied by increased tau phosphorylation in HN-h cells after HG stimulation, and ALKBH5 overexpression effectively reversed tau hyperphosphorylation. This is the first study to report that HG causes tau hyperphosphorylation by downregulating ALKBH5.

Tau is a substrate for many protein kinases, and tau phosphorylation is regulated by many protein kinases as well as other proteins [46]. As ALKBH5 is an m^6^A-modifying demethylase, ALKBH5-mediated demethylation activity affects nuclear RNA export and RNA metabolism and consequently regulates protein expression [47, 48]. Accordingly, we identified target proteins of ALKBH5 that could regulate tau hyperphosphorylation by m^6^A-mRNA epitope transcriptome microarray and RNA-seq. Nine transcripts overlapped in the RNA-seq and epitranscriptomic microarray data. We discovered by data analysis and review of the literature that Dgkh, Clasp1, and Pde1b are linked to central nervous system disease; however, only Dgkh showed reduced levels in the case of diabetes and HG-treated HN-h cells. Further, we showed by MeRIP-PCR that HG causes an increase in the m^6^A methylation modification of Dgkh mRNA, which is reversed following ALKBH5 overexpression. Overexpression of ALKBH5 can also rescue the HG-induced decrease in Dgkh mRNA and protein levels. Together, these findings indicate that ALKBH5 may participate in the progression of tau phosphorylation by modulating Dgkh expression.

Furthermore, we explored the role of Dgkh in tau hyperphosphorylation. Dgkh is a diacylglycerol kinase that alters the activity of its substrate diacylglycerol (DAG) and other effectors that affect cellular function [49]. Various DGK isoforms have been shown to have a regulatory role in synaptic plasticity in the CA1 hippocampus [50]. Dgkh is highly expressed in the hippocampus, cerebellum, brain striatum, and other tissues [51]. Its expression increases during mouse brain development, implying that this kinase may play a part in later development. Moreover, Dgkh has significant effects on bipolar disorder and attention-deficit/hyperactivity disorder [52, 53]. However, the role of Dgkh in diabetic cognitive dysfunction and other neurodegenerative disorders is yet to be discovered. In vivo and in vitro, we found that Dgkh overexpression effectively reduced tau hyperphosphorylation but did not affect ALKBH5 expression levels. This evidence suggests that Dgkh is downstream of ALKBH5 and that HG decreases ALKBH5-mediated Dgkh, leading to tau hyperphosphorylation in hippocampal neurons and diabetic cognitive dysfunction.

Next, we focused on the downstream mechanism of Dgkh. Under physiological conditions, when Dgkh expression is lowered, a rise in DAG concentration activates PKC attached to the plasma membrane [31, 54]. Several studies have shown that activation of PKC and several of its isoforms can lead to tau hyperphosphorylation. (i) In the AD human brain and AD animal brain tissues, tau hyperphosphorylation was observed to accompany increased PKC phosphorylation [55]. (ii) Inhibition of PKC activity decreased tau hyperphosphorylation in rat hippocampus tissues [33] and improved working memory in elderly rats [56], and PKC inhibitor Ro31-8220 prevented tau-induced neuronal loss [57]. (iii) In human SH-SY-5Y cells, overactivation of PKC-α promotes tau hyperphosphorylation of PHF-1 [58]. Therefore, we hypothesized that Dgkh may induce tau hyperphosphorylation by activating PKC-α. We further confirmed this by showing that HG significantly increased PKC-α levels in HN-h cells. When HN-h cells were treated with Ro31-8220, a PKC inhibitor, the tau hyperphosphorylation caused by HG and/or Dgkh knockdown was diminished, but the expression of ALKBH5 and Dgkh was not affected. Based on the above results, we infer that under HG conditions, ALKBH5 targets Dgkh to activate PKC-α, leading to tau hyperphosphorylation.

**Fig. 8:**
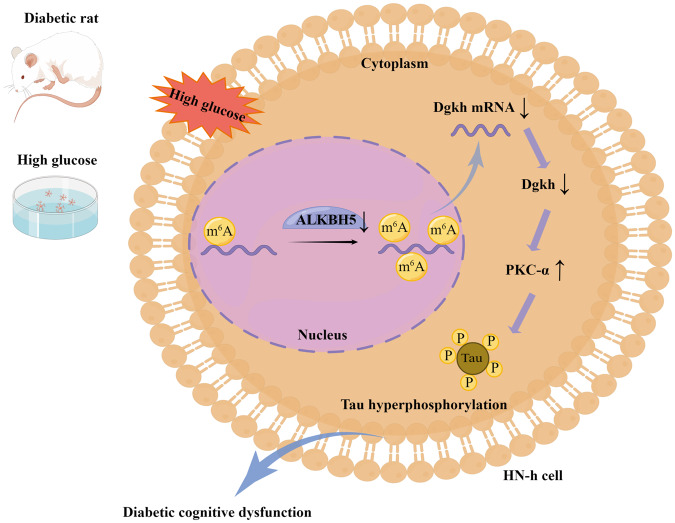
Proposed working model of the mechanism described in this study. High glucose suppresses the ALKBH5-mediated demethylation of Dgkh, which downregulates Dgkh and leads to tau hyperphosphorylation through activation of PKC-α in hippocampal neurons.

In conclusion, our findings demonstrate that HG inhibits the ALKBH5-mediated demethylation of Dgkh, which downregulates Dgkh and leads to tau hyperphosphorylation through activation of PKC-α in hippocampal neurons and diabetic cognitive dysfunction (Fig. 8). Our results may indicate a new mechanism of diabetic cognitive dysfunction and suggest new avenues for treatment strategies.

## Methods

### Antibodies

The following antibodies were used: rabbit monoclonal anti-GAPDH (Beyotime; AF1186; 1/1000); rabbit monoclonal anti-PHF1 (Abcam; ab184951; 1/1000); rabbit monoclonal anti-tau (phospho-T231; Abcam; ab151559; 1/1000); anti-tau (Affinity; AF6141; 1/1000); rabbit polyclonal anti-ALKBH5 (Proteintech; 16837-1-AP; 1/2000); mouse monoclonal anti-FTO (Abcam; ab92821; 1/1000); rabbit polyclonal anti-METTL3 (Proteintech; 15073-1-AP; 1/1000); rabbit polyclonal anti-METTL14 (ABclonal; A8530; 1/1000); rabbit polyclonal anti-Dgkh (Proteintech; 13873-1-AP; 1/1000); and anti-m^6^A (Synaptic Systems; 202,003).

### Streptozotocin-induced diabetic rat model and treatment

The Central South University Department of Laboratory Animals provided healthy male Sprague–Dawley (SD) rats (180–200 g) that had been reared under specific-pathogen-free conditions. After 1 week of adaptive feeding, the rats were randomly separated into diabetes and normal control groups. STZ (55 mg/kg, Sigma, St. Louis, MO, USA) was administered intraperitoneally to SD rats in the diabetic group to cause diabetes [59]. The glucose oxidase method was used to measure fasting plasma glucose levels (GOD-PAP; Boehringer Mannheim). Following 3 days of STZ injection, rats with blood glucose levels greater than 16.7 mmol/l were chosen for follow-up investigations [60]. The normal control and diabetic groups were divided into four groups at random for ventricular stereotaxic injection after the induction of diabetes for 10 weeks: (1) control rats stereotaxically injected with negative adenovirus (CON + AD-NULL); (2) diabetic rats stereotaxically injected with negative adenovirus (DM + AD-NULL); (3) diabetic rats stereotaxically injected with overexpression of Dgkh adenovirus (DM + AD-Dgkh); and (4) control rats stereotaxically injected with overexpression of Dgkh adenovirus (CON + AD-Dgkh). Behavioral experiments were performed at week 12, and the rats were sacrificed after the behavioral experiments had been completed.

### Injection of adenoviruses

Recombinant Dgkh adenovirus (HBAD-Adeasy-h-Dgkh-3xFLag-EGFP) and HBAD-EGFP-negative adenovirus suspensions were purchased from Hanheng Biotechnology (Shanghai) Co., Ltd. and stored at 80 °C until use. The titer of the adenovirus recombinant vector was 1.26×10^11^ titer units (TU)/ml. Adenovirus suspension was microinjected into the hippocampus CA1 regions as described previously [61]. According to the atlas of Paxinos and Watson (1998), a total volume of 2.0 μl adenovirus suspension was injected bilaterally into hippocampi using the following coordinates: anteroposterior±2.2 mm relative to bregma; lateral ±3.80 mm; dorsoventral 2.8 mm from the skull. Adenovirus suspensions were injected at a rate of 0.2 μl/min. The infusion needle was left in place for an additional 5 min to allow for diffusion.

### Morris water maze (WMW)

The spatial learning and memory abilities of rats were measured by the Morris water maze test. The water maze test was performed as described previously [62]. Smart v3.0 was used to track and record the rats’ movements (delay, distance, swimming speed, and navigation path). For training, also known as the hidden station test, a submerged platform (12 cm diameter) was placed in the center of the pool in the center of the southeast quadrant. The spatial exploration test was carried out 24 h after the sheltered station test and the station was removed. The residence time in the platform quadrant and the number of times the mice crossed the station quadrant were used to evaluate spatial memory ability.

### Cell culture

The HN-h cell line was obtained from Qingqi (Shanghai) Biotechnology Development Co. (BFN608007056). Cells were cultured in HG (25 mM) Dulbecco’s modified Eagle medium (Gibco C11965500BT, CA, USA) with 10% fetal bovine serum (Gibco 10099-141, CA, USA) and 1% penicillin/streptomycin solution (10,000 unit/mL penicillin and 10,000 μg/mL streptomycin; Gibco). As recommended by the manufacturer, cells were incubated at a constant temperature of 37 °C in an incubator containing 5% CO_2_. Digestion and passage were performed after the cells had reverted to a normal growth condition and reached 80–90% confluence. In this study, the following treatments were used: normal glucose (25 mM) and HG (50 mM glucose).

### Cell viability assay

HN-h cell viability was measured according to the protocol provided with the Cell Counting Kit-8 (CCK-8) kit (Dojindo, Japan). We inoculated HN-h cells into 96-well plates (2 × 10^4^ cells/ml) and treated them with different concentrations of glucose (25 mM, 33 mM, 50 mM, or 75 mM) or mannitol (33 mM, 50 mM, or 75 mM, plus 25 mM glucose in medium used as osmotic control) for varying durations (1 day, 2 days, or 3 days). The medium was replaced with 10% CCK-8 solution, followed by incubation at 37 °C for 2 h. The absorbance was then measured at 450 nm using a microplate reader (GENios, Tecan, Austria).

### Viral infection

HN-h cells were infected with Ad-ALKBH5/Ad-null (Shanghai Genechem Co., Ltd.) or Ad-Dgkh/Ad-null (Hanheng Biotechnology (Shanghai) Co., Ltd.). To confirm the overexpression of ALKBH5 and Dgkh, we collected cells 24 h after infection for RNA extraction and 48 h after infection for protein extraction. To investigate the potential impact of ALKBH5 and Dgkh overexpression on tau hyperphosphorylation, infected HN-h cells were exposed to HG after infection for 2 days.

### Transfection

HN-h cells were transfected with ALKBH5 siRNA, Dgkh siRNA, or SC siRNA (Hanheng Biotechnology (Shanghai) Co., Ltd.) following the manufacturer’s instructions, using Lipofectamine 2000 Transfection (Thermo; 11668027). Cells were taken at 24 h for RNA extraction and 48 h for protein extraction to determine the amount of ALKBH5 or Dgkh silencing. The cells were subsequently subjected to HG for 2 days after 48 h transfection to investigate the potential effect of ALKBH5 or Dgkh deletion on HG-induced tau hyperphosphorylation.

### RNA extraction and determination of RNA levels

Total RNA was extracted from cells and hippocampal tissue using TRIzol reagent (Invitrogen) following the manufacturer’s guidelines. For amplification, RNA was reverse transcribed into cDNA using a thermocycling method. The mRNA levels were measured at Applied Biosystems using SYBR Green dyes (TaKaRa, Beijing, China) for real-time quantitative PCR (qRT-PCR) analysis. The quantities of mRNA were normalized to β-actin. The primer sequences are shown in Table S1.

### m^6^A MeRIP-qRT-PCR

Total RNA was extracted from ALKBH5-overexpressing HN-h cells and matching control cells after HG stimulation. For RNA extraction, the cells were first lysed using a complete RIP lysis buffer (100 μl with 0.5 μl protease inhibitor cocktail and 0.25 μl RNase inhibitor for each reaction). RNA was then combined with 5 μg of either rabbit IgG or m^6^A antibody in 1 ml of RIP immunoprecipitation buffer (100 μl of the supernatant. m^6^A IP protease was incubated in proteinase K buffer (117 μl RIP wash buffer, 15 μl 10% SDS, 18 μl proteinase K for each experiment) at 55 °C for 30 min with shaking. Each sample was treated with 400 l phenol:chloroform:isoamyl alcohol in a 125:24:1 ratio for isolation of RNA before being subjected to m^6^A immunopurification and subsequent detection using qRT-PCR.

### Protein extraction, protein measurement, and western blot analysis

Cells and hippocampus tissues were homogenized and ruptured using a radioimmunoprecipitation assay buffer (NCM Biotech, WB3100, China) containing fresh protease and phosphatase inhibitors (TOPSCIENCE, China). Following protein denaturation, protein samples were separated by sodium dodecyl sulfate-polyacrylamide gel electrophoresis and then immunoblotted with antibodies. A ChemiDoc MP imaging system (Bio-Rad, USA) and Image Lab 6.0 system were used to perform blots using an enhanced chemiluminescence substrate kit (NCM Biotech, P2300, China).

### RNA-seq and data analysis

The extracted total RNA samples were submitted to agarose electrophoresis and NanoDrop quality control and quantification, and mRNA was enhanced with oligo(dT) magnetic beads. We built an RNA-seq library by a process including RNA fragmentation followed by inversion with random primers to generate first-strand cDNA, the addition of dUTP to synthesize second-strand cDNA, double-strand cDNA end repair plus A followed by ligation of Illumina matching junction, and PCR amplification to obtain the final library. The libraries were quality-validated using an Agilent 2100, quantified using the qPCR technique, and sequenced using an Illumina HiSeq 4000 sequencer. The sequenced data were subjected to quality control for quantitative gene and transcript expression analysis and other functional analyses.

### Rat m^6^A-mRNA epitranscriptomic microarray

Total RNA was extracted using TRIzol and quantified with a NanoDrop ND-1000 for each sample. An anti-m^6^A antibody was used to immunoprecipitate total RNA. The modified RNA (IP) was eluted from the immunoprecipitated magnetic beads, whereas the unmodified RNA (Sup) was recovered from the supernatant. We labeled the IP and Sup RNAs as cRNAs in separate procedures with Cy5 and Cy3, respectively, using an Arraystar Super RNA Labeling Kit. The cRNAs were combined and mixed with an Arraystar human mRNA and lncRNA epitranscriptomic microarray (8x60K, Arraystar) and hybridized. After the slides had been cleaned, the microarrays were scanned in a two-color channel using an Agilent Scanner G2505C. Agilent feature extraction software (version 11.0.1.1) was used to analyze the acquired array images. The raw intensities of the IP (Cy5-labeled) and Sup (Cy3-labeled) signals were normalized by the mean of the intensity of the internal reference Spike-in RNA on a log_2_ scale. After normalization, the signal levels of the probes were evaluated with respect to m^6^A methylation level and m^6^A quantity. The m^6^A methylation level as a percentage of modifications was calculated using the IP (Cy5-labeled) and Sup (Cy3-labeled) normalized intensities. The amount of m^6^A methylation was calculated using the IP (Cy5-labeled) normalized intensity. Filtering with ploidy change and statistical significance (*p*-value) thresholds were used to identify differential m^6^A-methylated RNAs between the two controls. To demonstrate distinguishable m^6^A-methylation patterns between samples, hierarchical clustering was used.

### Statistical analyses

Data were statistically analyzed using GraphPad Prism 8.0 software. All data were first examined by normality test and chi-square test. Student’s two-sample *t*-test was used to compare differences between two samples satisfying a normal distribution according to the chi-square test, and Mann–Whitney test was used for non-normal distributions. One-way analysis of variance and Tukey–Kramer test were used for comparisons of differences among several samples satisfying a normal distribution according to the chi-square test, and the Kruskal–Wallis *H* non-parametric test was used for non-normally distributed samples. Data are presented as mean ± SEM, as indicated in the figure legends. All experiments were performed independently three times. *p* < 0.05 was regarded to indicate a statistically significant difference.

## Supplementary information

Supplement figure legends

Figure S1

Figure S2

Figure S3

Table S1

## Data Availability

All data generated or analyzed during this study are included in this published article. Raw and processed data are stored in the GEO database and are available upon request.
