# Supplementary material for: High glucose induces tau hyperphosphorylation in hippocampal neurons via inhibition of ALKBH5-mediated Dgkh m6A demethylation: a potential mechanism for diabetic cognitive dysfunction
Source: Cell Death Dis. 2023 Jun 29;14(6):385. doi: 10.1038/s41419-023-05909-7 (PMC10310746; doi:10.1038/s41419-023-05909-7)
Supplement: Supplementary file 1 — Supplement figure legends [file 41419_2023_5909_MOESM1_ESM.docx]

**Figure S1: Expression of m^6^A-related enzymes in the diabetic hippocampus.** (A) Real-time quantitative PCR analysis of METTL3, METTL14, FTO, and ALKBH5 mRNA expression in diabetic rats (n=4). (B) Western blots and quantitative densitometry analysis of METTL3, METTL14, FTO, and ALKBH5 in diabetic rats (n=3). Student’s two-sample t-test and Mann–Whitney test were used to detect differences between the two groups. *p<0.05, **p<0.01 vs CON. Error bars represent s.e.m.

**Figure S2: Expression of m^6^A-related enzymes in HN-h cells.** (A) Real-time quantitative PCR analysis of METTL3, METTL14, FTO, and ALKBH5 mRNA expression in HN-h cells with 50 mM glucose (n=3). (B) Western blots and quantitative densitometry analysis of METTL3, METTL14, FTO, and ALKBH5 in HN-h cells with 50 mM glucose (n=3). Student’s two-sample t-test and Mann–Whitney test were used to detect differences between the two groups. *p<0.05, **p<0.01 vs CON. Error bars represent s.e.m.

**Figure S3: Expression of PKC-α in the diabetic hippocampus.** (A) Western blots and quantitative densitometry analysis of PKC-α in diabetic rats (n=3). Student’s two-sample t-test and Mann–Whitney test were used to detect differences between the two groups. **p<0.01 vs CON. (B) Western blots and quantitative densitometry analysis of PKC-α in diabetic rats with adenovirus-mediated overexpression of Dgkh (n=3). One-way ANOVA and Tukey–Kramer test were used to detect differences among groups. **p<0.01 vs CON+AD-NULL; ^##^p<0.01 vs DM+AD-NULL. Error bars represent s.e.m.
