# Supplementary figures and images for: High glucose induces tau hyperphosphorylation in hippocampal neurons via inhibition of ALKBH5-mediated Dgkh m6A demethylation: a potential mechanism for diabetic cognitive dysfunction
Source: Cell Death Dis. 2023 Jun 29;14(6):385. doi: 10.1038/s41419-023-05909-7 (PMC10310746; doi:10.1038/s41419-023-05909-7)

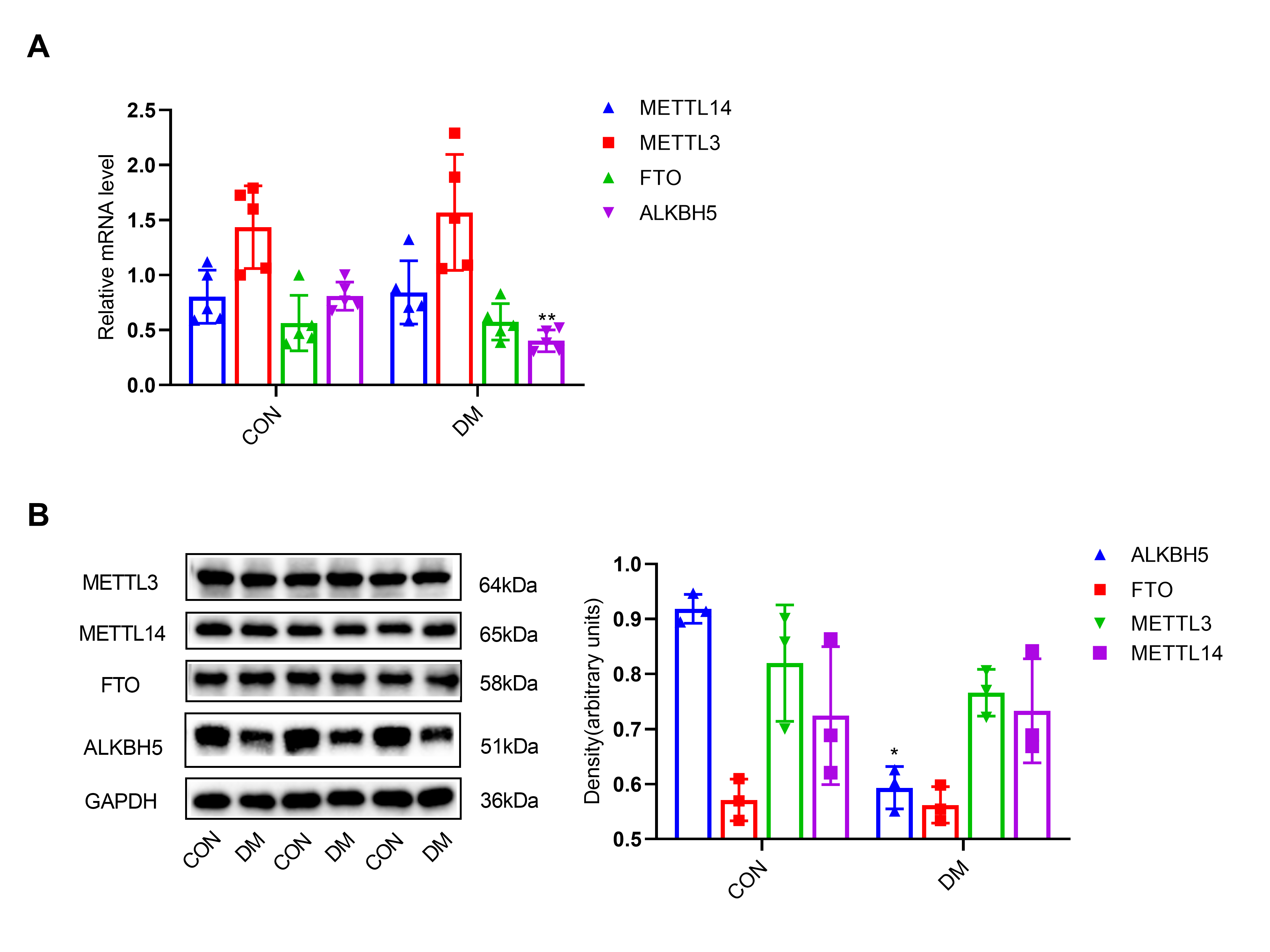

Supplement: Supplementary file 2 — Figure S1 [file 41419_2023_5909_MOESM2_ESM.tif]

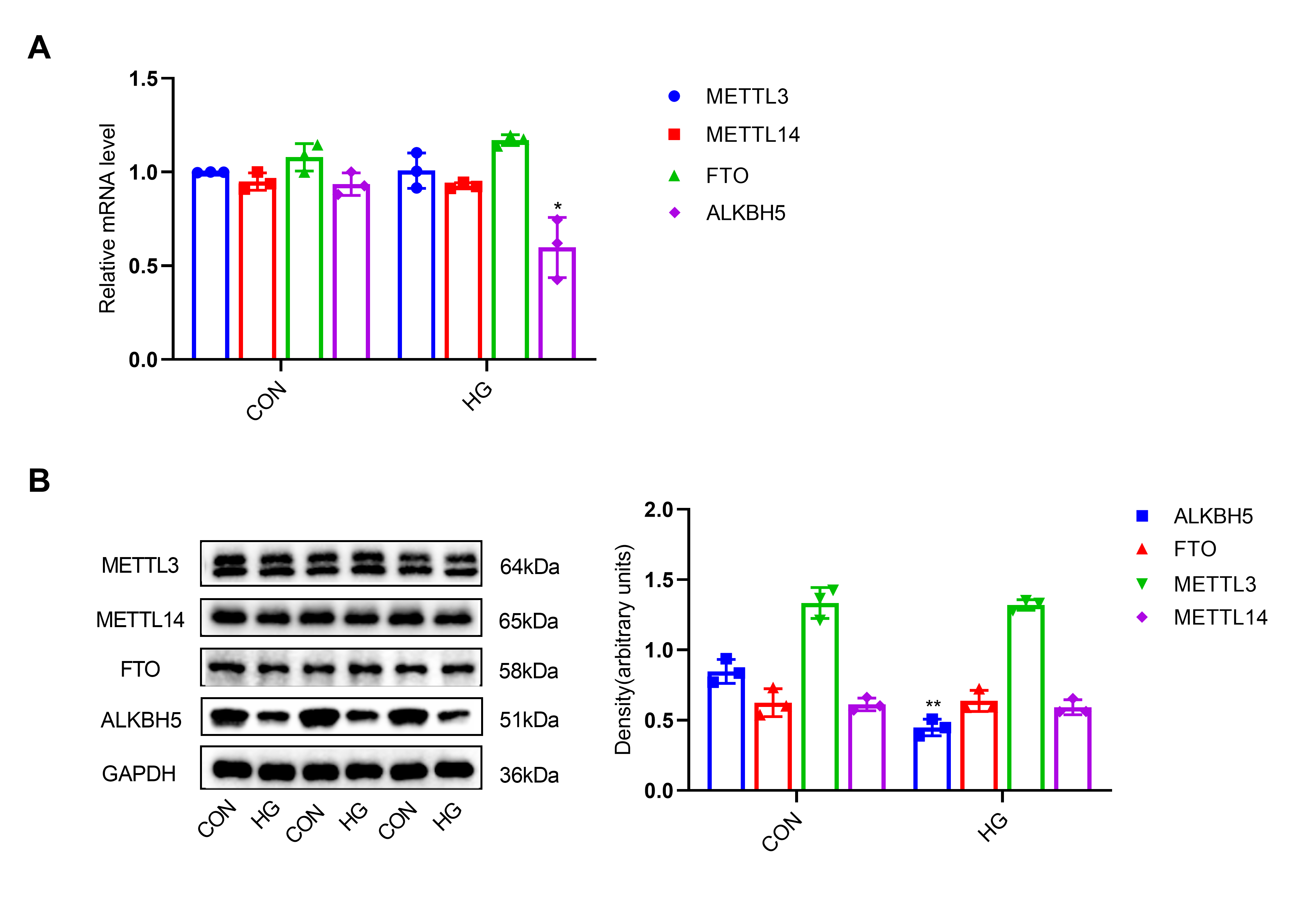

Supplement: Supplementary file 3 — Figure S2 [file 41419_2023_5909_MOESM3_ESM.tif]

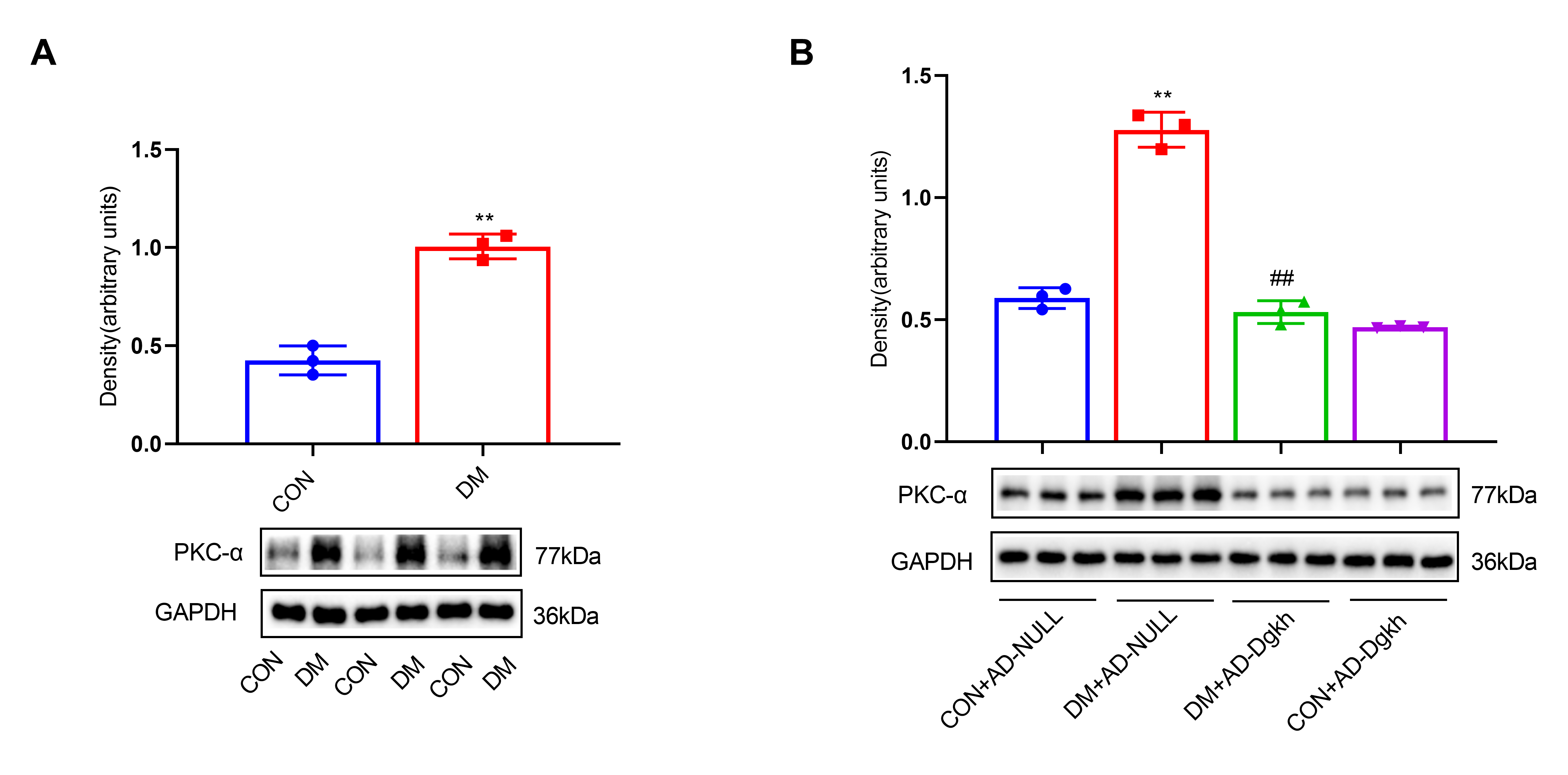

Supplement: Supplementary file 4 — Figure S3 [file 41419_2023_5909_MOESM4_ESM.tif]
