# Supplementary material for: High glucose induces tau hyperphosphorylation in hippocampal neurons via inhibition of ALKBH5-mediated Dgkh m6A demethylation: a potential mechanism for diabetic cognitive dysfunction
Source: Cell Death Dis. 2023 Jun 29;14(6):385. doi: 10.1038/s41419-023-05909-7 (PMC10310746; doi:10.1038/s41419-023-05909-7)
Supplement: Supplementary file 5 — Table S1 [file 41419_2023_5909_MOESM5_ESM.docx]

Table S1: Primers

| Species | mRNA target | Sense/anti-sense |
| --- | --- | --- |
| Rat | ALKBH5 | F: 5'-ACCCCATCCACATCTTCGAG-3’  R: 5'-CATCAGCAGCATACCCACTGAG-3’ |
|  | FTO | F: 5'-GATCCACAACGAGGTCGAGT-3  R: 5'-TGCTGTGCTGGTAGAGTTCG-3’ |
|  | METTL3 | F: 5'-AGCTGAGGTTCGTTCCACCA-3’  R: 5'-GCCACGGCTCTCAATGTCTC-3’ |
|  | METTL14 | F: 5'-CCTTCCTTAAGGGAACGCAG-3’  R: 5'-GATGCCGGTCTCTCTGTAGT-3’ |
|  | Dgkh | F: 5'-TGATGTGGTATGGCGTCCTT-3'  R: 5'-CGGTGATGCTGCAATTTGAT-3’ |
|  | β-ACTIN | F: 5'-CGAGTACAACCTTCTTGCAGC-3’  R: 5'- ACCCATACCCACCATCACAC-3’ |
| Homo | ALKBH5 | F: 5'-CCTTGGTTTTGTTGCCTGT-3’  R: 5'-TCTGTCCCCTATTGATGCC-3’ |
|  | FTO | F: 5'-ACGAATTGCCCGAACAT-3  R: 5'-ATCCTTTTCCCAGTATGGC-3’ |
|  | METTL3 | F: 5'-TTGCCCACTGATGCTGT-3  R: 5'-GGAGACCTCGCTTTACCTC-3’ |
|  | METTL14 | F: 5'-CCAACGCTTACAAATAGCAAC -3  R: 5'-CCTCCTCGGTCAGATTTAGA-3’ |
|  | Dgkh | F: 5'-CATCATACAAACCCTCACCA-3'  R: 5'-CGGTGATGCTGCAATTTGAT-3’ |
|  | β-ACTIN | F: 5'-TCTCACGCCCCTTATTGCT-3’  R: 5'-CCTCTCGTCCTCTCTCCCA-3’ |
